# The Stigma of Migraine

**DOI:** 10.1371/journal.pone.0054074

**Published:** 2013-01-16

**Authors:** William B. Young, Jung E. Park, Iris X. Tian, Joanna Kempner

**Affiliations:** 1 Department of Neurology, Thomas Jefferson University, Philadelphia, Pennsylvania, United States of America; 2 Department of Sociology, Rutgers University, New Brunswick, New Jersey, United States of America; Institute of Neuroepidemiology and Tropical Neurology, France

## Abstract

**Background:**

People who have a disease often experience stigma, a socially and culturally embedded process through which individuals experience stereotyping, devaluation, and discrimination. Stigma has great impact on quality of life, behavior, and life chances. We do not know whether or not migraine is stigmatizing.

**Methods:**

We studied 123 episodic migraine patients, 123 chronic migraine patients, and 62 epilepsy patients in a clinical setting to investigate the extent to which stigma attaches to migraine, using epilepsy as a comparison. We used the stigma scale for chronic illness, a 24-item questionnaire suitable for studying chronic neurologic diseases, and various disease impact measures.

**Results:**

Patients with chronic migraine had higher scores (54.0±20.2) on the stigma scale for chronic illness than either episodic migraine (41.7±14.8) or epilepsy patients (44.6±16.3) (p<0.001). Subjects with migraine reported greater inability to work than epilepsy subjects. Stigma correlated most strongly with the mental component score of the short form of the medical outcomes health survey (SF-12), then with ability to work and migraine disability score for chronic and episodic migraine and the Liverpool impact on epilepsy scale for epilepsy. Analysis of covariance showed adjusted scores for the stigma scale for chronic illness were similar for chronic migraine (49.3; 95% confidence interval, 46.2 to 52.4) and epilepsy (46.5; 95% confidence interval, 41.6 to 51.6), and lower for episodic migraine (43.7; 95% confidence interval, 40.9 to 46.6). Ability to work was the strongest predictor of stigma as measured by the stigma scale for chronic illness.

**Conclusion:**

In our model, adjusted stigma was similar for chronic migraine and epilepsy, which were greater than for episodic migraine. Stigma correlated most strongly with inability to work, and was greater for chronic migraine than epilepsy or episodic migraine because chronic migraine patients had less ability to work.

## Introduction

Stigma is an established construct in the social sciences that describes a characteristic, trait, or diagnosis that discredits individuals and elicits prejudice, discrimination, and loss of status. Goffman characterized stigma as a process that spoils the identity of the stigmatized individual [1]. Many diseases, such as HIV, depression, and epilepsy, are known to be stigmatizing and result in disruption of social relationships, decreased quality of life, and loss of employment [2]–[4]. While being stigmatized is in itself a negative consequence of disease, it also has health implications, because it affects the way individuals experiencing stigma seek and access medical care, and because the lack of social belonging is stressful and incurs negative health outcomes [5]–[7].

Stigma attaches to disease to varying degrees and in various ways. Stigma can be “enacted,” as when individuals actually experience discrimination, for example, through the loss of a social relationship or employment. Stigma may also be “internalized” or perceived, which refers to individual’s own feelings about their condition, including anticipation about how others might react to it [8]. Subjective experiences of stigma can be as damaging to health as acts of discrimination and the actual loss of social relationships [9]. Although many claim that migraine is stigmatizing, to date there has been only one study on stigma in people with migraine [10]. In our study we measure how much stigma migraine patients experience in comparison to epilepsy patients–a group that has been studied extensively [11].

## Methods

### Study Population

Between October 2009 and July 2011, we recruited patients with migraine or epilepsy from the Jefferson Headache Center and the Jefferson Comprehensive Epilepsy Center in Philadelphia, Pennsylvania. Questionnaires were administered in an office setting. Inclusion criteria were subjects between the ages of 18–65, who had migraine or epilepsy diagnosed by a specialist. Exclusion criteria were inability to give accurate responses and a diagnosis of epilepsy and severe episodic migraine (EM) or chronic migraine (CM). Migraine is a primary headache disorder with attacks lasting 4 to 72 hours untreated and at least two of the following features: severe pain, unilaterality, throbbing, or exacerbation with activity; and one of the following features: nausea or light and sound sensitivity. The migraine type was determined by an attending physician who was board certified in headache medicine, following criteria set forth in the International Classification of Headache Disorders [12]. If the number of headache days per month is greater than 14, and eight of these headache days met criteria for migraine (or would if not treated), then the person had CM. If the number of headache days per month is 14 or less, then the person had EM.

The institutional review board at Thomas Jefferson University Hospital reviewed and approved this study, and written informed consent was obtained from each participant.

### Questionnaire Administration

Both migraine and epilepsy patients completed a demographic questionnaire, the stigma scale for chronic illness (SSCI), and the short form of the medical outcomes health survey (SF-12). They also completed a rankable series of questions on their actual or potential (if they should try to get a job) disability. Migraine patients completed the migraine disability score (MIDAS) and answered a rankable series of questions on the degree of resting necessitated by their headaches, and epilepsy patients completed the Liverpool impact of epilepsy scale.

### Stigma Scale for Chronic Illness (SSCI)

The SSCI is a 24-item questionnaire that quantifies the degree and impact of stigma in patients with chronic illnesses. Thirteen items measure “self/internalized stigma,” asking, for example, whether the subject feels a sense of shame or anxiety about their condition (SSCI-I) and 11 measure “enacted stigma,” asking, for example, about instances of actual discrimination (SSCI-E) [13], [14].

### SF-12 Health Survey

The SF-12 is a subset of the SF-36 quality-of-life questionnaire that is used for patient-based assessments of physical and mental health.

### MIDAS

The MIDAS is a brief questionnaire that is used to quantify the disability of migraine over a 3-month period. The number of days the patient had migraine in the last 90 days and the average headache severity are also assessed.

### The Liverpool Impact of Epilepsy Scale

This scale is a 9-item component of the extensive Liverpool Seizure Severity Scale that assesses patients’ perception of the impact of epilepsy on work, activity, personal relationships, and self-image.

### Ability to Work Score

**Figure 1:**
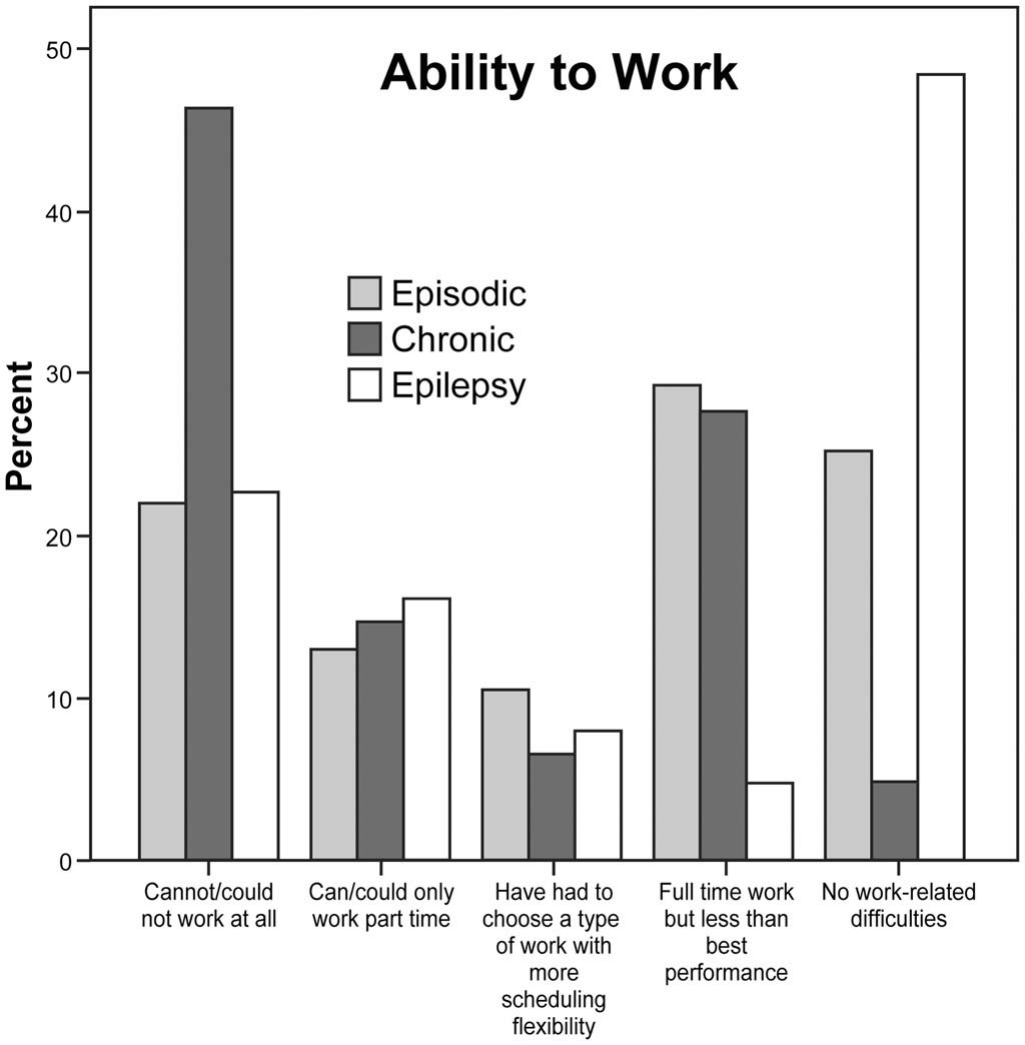
Effect on Work. Results of questionnaire in which subjects were asked about whether they could work. If they were not working and not trying to work they were asked to imagine trying to work with their current headache or epilepsy condition. Chronic migraine had worse ability to function.

Subjects were asked about the impact migraine or epilepsy had on their ability to work (and to assume they were trying to work if they were homemakers, retired, or had given up trying to work) using five ranked questions (the lowest level of function was chosen if multiple responses were given) (Figure 1).

### Need to Rest Score

**Figure 2:**
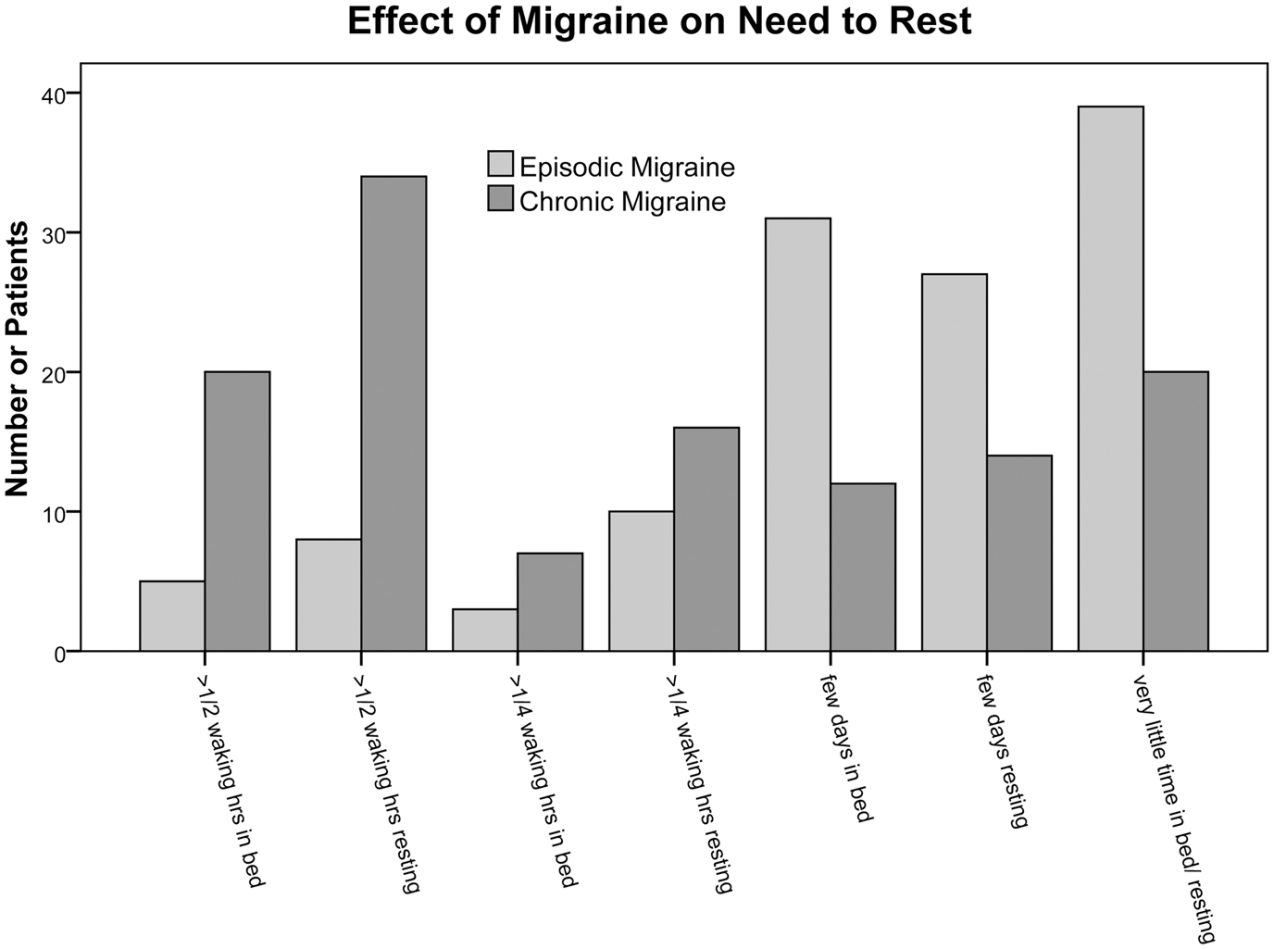
Effect on Need to Rest. Distribution of answers to questionnaire on the effect of migraine on need to rest and spending time in bed. Subjects with chronic migraine reported spending more time in bed and resting (p<0.001, Krushal-Wallis test).

Subjects with migraine were asked about their need to lie down or rest. They were given seven possible answers (Figure 2) that were ranked with the lowest level of function if multiple responses were given.

### Statistical Considerations

To detect a difference of 10 points in the SSCI score between the more and less disabled halves of the EM and CM patients at a 0.05 significance level, assuming a standard deviation of 19.7 (based on previous data), a sample size of 62 was required [13]. Thus, 124 subjects were required for each group. To distinguish a 10-point difference in mean scores between Ep and CM patients, a sample of 62 patients would be sufficient. Data were analyzed using SPSS. Pearson correlations were used unless otherwise specified.

## Results

One hundred twenty-four EM and CM and 62 Ep patients completed the study. One subject in each migraine group was excluded due to incomplete data, other than income. Internal consistency for the four scales, as assessed by Cronbach’s alpha, achieved good levels. The coefficients for the four scales were 0.96, 0.74, 0.86, and 0.83 for SSCI, MIDAS, Liverpool Impact Scale, and SF-12, respectively.

A higher percentage of EM and CM than Ep patients were female. EM patients were slightly older and had more education and higher incomes (Table 1). CM patients had more work-related disability (or potential disability) than EM patients and Ep patients (p<0.001 EM vs CM, and Ep vs CM, p = NS EM vs Ep Mann-Whitney) (Figure 1). Headache severity was similar in migraine patients (Table 2). Patients with CM had greater need to rest than patients with EM (Figure 2). Of the 62 epilepsy patients, 21 patients (33.9%) had a history of surgery (i.e., temporal lobectomy or resection of the seizure foci), two patients (3.2%) had a history of both surgery and vagus nerve stimulator insertion, one patient (1.6%) had a history of vagus nerve stimulator insertion, and 38 patients (61.3%) did not have any history of surgery or vagus nerve stimulation. Epilepsy was focal in 49 patients (79%), generalized in nine (14.5%) and unspecified in four (6.5%). Forty-eight patients (77.4%) did not report any generalized tonic-clonic seizures in the past month, while 12 patients (19.4%) did. One-year seizure frequencies (which included all types of seizures) were divided into three groups; 25 patients were seizure-free (40.3%), 25 patients (40.3%) had less than four seizures, and 10 (16.1%) patients had more than four seizures.

**Table 1:** Basic Demographics.

	EpisodicMigraine(EM)(n = 123)	ChronicMigraine(CM)(n = 123)	Epilepsy(Ep)N = 62	p Value	CM/EMp	EM/EpP	CM/EpP
Age	44.7±12.5	40.9±12.2	38.4±13.1	0.003*	0.054**	0.054**	0.004**
Gender					NS°	<0.001°	<0.001°
Male	17.1%	15.4%	45.2%				
Female	82.9%	84.6%	59.8%				
Race				0.028†	0.036††	0.008††	NS††
Caucasian	95.1%	87.8%	83.9%				
African-American	4.1%	7.3%	6.5%				
Other	0.8%	4.9%	6.5%				
Education				<0.001†	0.015††	<0.001††	0.009††
High school	15.4%	17%	35.5%				
Some college	12.2%	24.4%	21.0%				
Associates	5.7%	8.9%	11.3%				
Bachelors	31.7%	26.8%	17.7%				
Graduate/Professional	35.0%	22.8%	14.5%				
Household Income				<0.001†	<0.001††	<0.001††	0.21††
0–18.5000	5.7%	13.8%	21.0%				
18,5005–35000	8.9%	11.4%	19.4%				
35,000–55,000	9.8%	21.1%	14.5%				
55,000–90,000	28.5%	31.7%	14.5%				
>90,000	46.3%	20.3%	24.2%				
N	122	121	58				

* = ANOVA;

** multiple comparisons with Bonferroni adjustment.

° = Chi Square test.

† = Kruskal-Wallis;

†† = Mann-Whitney.

**Table 2:** Measurements of Stigma, Quality of Life, and Impact.

	EpisodicMigraine	ChronicMigraine	Epilepsy	p* value	CM/EMp	CM/Epp	EM/Epp
Total SSCI score	41.7±14.8(Median: 36)	54.0±20.2(Median: 53)	44.6±16.3(Median: 42.5)	<0.001*	<0.001	0.002	NS
SSCI –I	26.1±10.0(Median: 24)	34.5±12.9(Median: 33)	26.7±9.7(Median: 28)	<0.001*	<0.001	<0.001	NS
SSCI –E	14.9±5.8(Median: 13)	19.5±8.3(Median: 17)	18.0±8.1(Median: 14)	<0.001*	<0.001	<0.001	NS
SSCI-I/SSCI Total	0.622±0.003	0.637±0.004	0.598±0.005	<0.001	NS	<0.001	0.049
SF-12							
PCS	42.31±8.5(Median: 43)	37.1±8.1(Median: 37)	47.6±9.4(Median: 51)	<0.001*	<0.001	<0.001	<0.001
MCS	47.61±10.2(Median: 49)	39.4±11.8(Median: 37)	46.6±11.9(Median: 48)	<0.001*	<0.001	<0.001	NS
Total MIDAS score	28.54±38.7(Median: 19)	86.5±77.86(Median: 60)			<0.001†		
Headache frequency (per 90 days)	18.7±13.3(Median: 16)	55.2±29.1(Median: 60)			<0.001†		
Severity (0–10 scale)	6.1±3.6(Median: 6.0)	6.3±1.4(Median: 6.0)			0.085†		
Liverpool Impact			9.2±6.8(Median: 8)				

* ANOVA, Bonferroni correction for multiple comparisons;

† Mann-Whitney. Headache severity and frequency based on MIDAS questions 6 and 7.

**Abbreviations:** SCCI = stigma scale for chronic illness; SSCI-I = internalized stigma; SSCI-E = enacted stigma; SF-12 = short form 12 in the medical outcomes study Quality of Life questionnaire; PCS = physical component subscale of SF-12; MCS = mental component subscale of SF-12; MIDAS = migraine disability scale; Liverpool impact scale = component of the Liverpool seizure severity scale.

**Figure 3:**
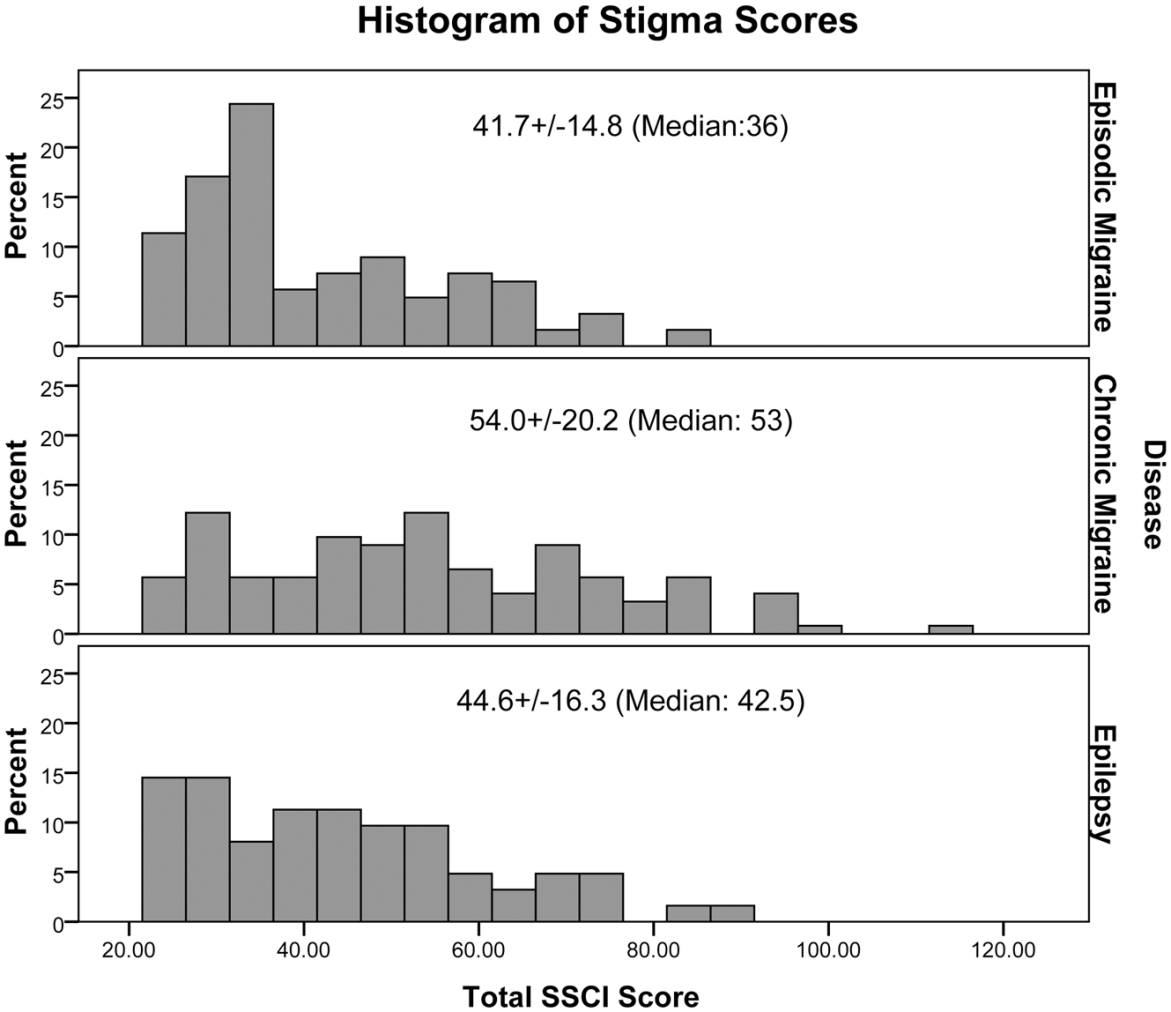
Stigma Scores. Distribution of stigma scores for EM, CM, and Ep. Scores for CM are significantly higher than for EM or Ep. CM scores were higher than SSCI scores of 42.7+/−19.7 for an internet panel of diverse neurological patients used to validate the SSCI (p<0.001, z-test), while EM and Ep were not.

CM patients experienced statistically significantly more stigma than EM or Ep patients as measured by SSCI scores (Figure 3, Table 2). We examined the ratio of internalized stigma to the total stigma score, SSCI-I/SSCI, and found that a higher proportion of the stigma reported by CM and EM patients could be attributed to internalized stigma than enacted stigma (Table 2). The ratio of SSCI-I to SSCI (total) did not vary by age or gender, but correlated negatively with ability to work (r = −0.201, p<0.001). Functional and quality-of-life measurements were lower for CM than EM (Table 2). SF-12 scores were lower for CM than EM or Ep patients, and lower for EM than Ep patients on the physical component subscale (PCS) but not the mental component subscale (MCS) (Table 2).

### Correlations

SSCI, SSCI-I, and SSCI-E were strongly correlated (SSCI with SSCI-E, and SSCI-I; r = 0.904, and 0.963, and for SSCI-E with SSCI-I r = 0.755, p<0.001 for all) and with similar correlations for each disease group. In general, correlations for SSCI, SSCI-I, and SSCI-E were similar. Therefore, we will present only correlations with SSCI and indicate areas where results differed between SSCI-I and SSCI-E (Table 3).

**Table 3:** Correlations Between Scales.

Migraine Type			SSCI-I	SSCI-E	Ability to work	PCS12	MCS12	Total MIDAS	Need to rest	Total Impact
**Episodic** **(N = 123) A**	Total SSCI	p-value	.965	.889	−.511	−.179	−.592	.445	−.464	
		Sig. (2-tailed)	.000	.000	.000	.047	.000	.000	.000	
	SSCI int	p-value		.738	−.531	−.198	−.636	.471	−.498	
		Sig. (2-tailed)		.000	.000	.028	.000	.000	.000	
	SSCI enact	p-value			−.389	−.116	−.415	.324	−.324	
		Sig. (2-tailed)			.000	.202	.000	.000	.000	
	Ability to work	p-value				.336	.328	−.366	.490	
		Sig. (2-tailed)				.000	.000	.000	.000	
	PCS12	p-value					−.018	−.258	.281	
		Sig. (2-tailed)					.845	.004	.002	
	MCS12	p-value						−.497	.427	
		Sig. (2-tailed)						.000	.000	
	Total MIDAS	p-value							−.407	
		Sig. (2-tailed)							.000	
	Need to rest	p-value								
		Sig. (2-tailed)								
**Chronic** **(N = 123) B**	Total SSCI	p-value	.968	.919	−.497	−.429	−.575	.405	−.429	
		Sig. (2-tailed)	.000	.000	.000	.000	.000	.000	.000	
	SSCI int	p-value		.789	−.511	−.412	−.616	.433	−.449	
		Sig. (2-tailed)		.000	.000	.000	.000	.000	.000	
	SSCI enact	p-value			−.408	−.398	−.434	.308	−.340	
		Sig. (2-tailed)			.000	.000	.000	.001	.000	
	Ability to work	p-value				.469	.321	−.368	.577	
		Sig. (2-tailed)				.000	.000	.000	.000	
	PCS12	p-value					.022	−.349	.349	
		Sig. (2-tailed)					.808	.000	.000	
	MCS12	p-value						−.332	.430	
		Sig. (2-tailed)						.000	.000	
	Total MIDAS	p-value							−.548	
		Sig. (2-tailed)							.000	
	Need to rest	p-value								
		Sig. (2-tailed)								
**Epilepsy** **(N = 62) C**	Total SSCI	p-value	.932	.901	−.475	−.472	−.581			.527
		Sig. (2-tailed)	.000	.000	.000	.000	.000			.000
	SSCI int	p-value		.684						.530
		Sig. (2-tailed)		.000	−.461	−.443	−.594			.000
	SSCI enact	p-value			−.407	−.423	−.461			.428
		Sig. (2-tailed)			.001	.001	.000			.001
	Ability to work	p-value				.521	.362			−.452
		Sig. (2-tailed)				.000	.004			.000
	PCS12	p-value					.444			−.533
		Sig. (2-tailed)					.000			.000
	MCS12	p-value								−.740
		Sig. (2-tailed)								.000
	Total MIDAS	p-value								
		Sig. (2-tailed)								
	Need to rest	p-value								
		Sig. (2-tailed)								

2-tailed Pearson correlation coefficients for variables associated with episodic (**A**) and chronic (**B**) migraine, and for epilepsy (**C**). The p-values are not adjusted for multiple comparisons.

**Abbreviations:** SCCI = stigma scale for chronic illness; SSCI-I = internalized stigma; SSCI-E = enacted stigma; PCS = physical component subscale of SF-12; MCS = mental component subscale of SF-12; MIDAS = migraine disability scale.

All three groups demonstrated no association of gender or race on SSCI, although the groups were heavily white. Migraine groups showed no effect of age, education, or income on SSCI. With epilepsy only, SSCI correlated positively with age (r = 0.347, p = 0.006) and negatively with education (Spearman’s r = −0.346, p = 0.008) and income (r = −0.336, p = −0.008). All groups had a strong negative correlation between ability to work and SSCI (r = −0.511 (EM), r = −0.497 (CM), r = −0.475 (Ep), p<0.001 for all). SSCI was negatively correlated with PCS (r = −0.179 p = 0.047 (EM), r = −0.429 p<0.001 (CM), and r = −0.472 p<0.001 (Ep )) and MCS (r = −0.592 p<0.001 (EM), r = −0.575 p<0.001 (CM) and r = −0.581 p<0.001 (Ep)). Only for EM did the SSCI-E not correlate with PCS (Table 3). In migraine, disability (MIDAS) was correlated with SSCI for EM (r = 0.445) and for CM (0.405, p<0.001 for both). In Ep, impact score correlated with SSCI Score (r = 0.527, p<0.001). MIDAS and ability to work were only modestly correlated (r = −0.366 for EM, r = −0.368 for CM, p<0.001 for both), indicating substantial differences in what these variables measured.

In migraine, SSCI had a moderate negative correlation with need to rest (Spearman’s r = 0.479, p<0.001 (EM) 0.400, p<0.001 (CM)). SSCI correlated with headache severity for EM (r = 0.205, p = 0.023) and CM (r = 0.218, p = 0.015). SSCI-E did not correlate with severity for EM (r = 0.033, p = NS), but did for CM (r = 0.211, p = 0.019). Migraine frequency did not correlate with SSCI for EM (r = 0.086, p = NS) but did for CM (r = 0.218, p = 0.015).

### Analysis of Covariance (ANCOVA)

A 2 (Gender) X 3 (Group: EM, CM, and Ep) ANCOVA was conducted. The dependent variable was the SSCI score and the covariates were: (a) age, (b) income, (c) ability to work, (d) physical component score (SF12), and (e) education. Preliminary analyses evaluating the homogeneity-of-slopes assumption indicated that the relationships between the covariates and the dependent variable did not differ significantly. Results indicated no statistically significant interaction effect or gender effect; however, there was a significant group effect: F (2, 290) = 3.17, p = .043, η^2^ = .021. Follow-up tests were conducted to evaluate pairwise differences among these adjusted means. Based on the least significant difference procedure, the adjusted mean for EM (43.7; 95% confidence interval, 40.9 to 46.6) differed significantly from both CM (49.3; 95% confidence interval, 46.2 to 52.4) and Ep (46.5; 95% confidence interval, 41.6 to 51.6). There were no significant differences between CM and Ep. Ability to work achieved a strong association with SSCI as measured by the η^2^ of.157. The only other significant covariant was PCS with a weak η^2^ of.024.

## Discussion

In this study we used the SSCI, a newly developed questionnaire to assess stigma in multiple neurological illnesses. The SSCI was developed as one element of a multiple site project to produce relevant and psychometrically robust quality of life assessment tools for adults and children. The SSCI is one of 13 tools resulting from this project and is available as a 24-item questionnaire, as well as an 8-item short form, which was not yet available when the study was conducted. The 24-item scale has two subscales, “internalized” and “enacted”. Stigma scores for Ep and EM were consonant with those found in a panel of diverse neurological patients whose SSCI scores averaged 42.7 (+/−19.7). CM patients reported much higher SSCI scores than either group (54.0+/−20.2). CM patients reported particularly high SSCI-I scores, which suggests that, of the three groups, they are the most likely to identify with the stereotypes and negative labels that attach to migraine.

Our finding that CM patients reported more stigma than both epilepsy patients and patients with EM contradicts that of Aydemir et. al, who found that epilepsy patients felt more stigmatized than migraine patients in a clinical sample in Turkey [10]. Methodological differences may explain these divergent findings. Aydemir et. al did not differentiate between CM and EM, which, according to our analysis, could affect stigma scores. In addition, they used a three-item stigma scale that had not been validated for use across disease groups. Finally, stigma is a process deeply embedded in social and cultural norms, and stigma might attach differently to epilepsy and migraine in Turkey than in the United States.

We observed a numerically greater SSCI-I than SSCI-E for the migraine groups, so we created an exploratory variable, SSCI-I/Total SSCI to assess internalized stigma as a proportion of the overall stigma score. Although the mean ratio of each group differed only slightly, the standard deviation was small and the difference reached significance. Both CM and EM report higher rates of internalized stigma as a proportion of their overall stigma score than Ep. This suggests that migraine patients have a more vigorous process of converting enacted stigma into internalized stigma. Alternatively, migraine patients may be better able to suppress enacted stigma by being more circumspect about divulging their medical condition while experiencing the internalized stigma commensurate with their illness severity [15].

Migraine patients reported equally high stigma scores across age, income, and education. In contrast, for epilepsy patients, younger age, higher education, and higher income correlated with lower SSCI scores. This finding suggests that a cultural shift may be underway and that education and anti-stigma efforts in epilepsy are taking hold. Anti-stigma efforts have been more limited for migraine.

Patients’ reports of the impact of their disease correlated with SSCI scores for both migraine (as measured by MIDAS, ability to work and need to rest) and epilepsy (as measured by the Liverpool impact scale). The only exception to this was pain severity, which correlated with SSCI for CM, but not for SSCI-E in EM. This may indicate more reserve among patients with EM to handle more severe headache pain, or it may suggest that experiencing intermittent, severe head pain, as people with EM do, is less stigmatizing, since it is more consistent with public perception of normal migraine.

The mental component of the SF-12 was more highly correlated with stigma than the physical component. In our analysis, we elected to model the physical component score of the SF-12 as an independent variable as it seems implausible that the physical consequences of migraine or epilepsy were caused by stigma. A more difficult choice was to view the MCS as an independent variable and exclude it from the analysis. This is consistent with the proposition that the stigma itself causes the MCS change, and seems more plausible than higher MCS causing stigma. This assumption should be viewed with some skepticism as an increased MCS as a result of migraine or its comorbidities could result in increased stigma and elevation of the SSCI score.

In our model, once we accounted for all factors, CM and Ep had similar stigma while EM had less. In other words, CM incurs more stigma than Ep, but only because in our sample people with CM experience more disability and are less able to work. However, CM incurs more stigma than EM, even when all factors are taken into account, perhaps because EM aligns best with public perceptions of the disease. Although we were able to find significant correlations, we were only able to account for a modest amount of the variance, indicating that other factors not considered here, including individual factors, such as resilience, are likely important determinants of an individual’s experience of stigma.

The study has several strengths. It is the largest study of stigma in migraine; the first study of stigma in migraine that uses validated scales, including the SSCI; and the first study of stigma in migraine in the United States. In this case, the reliance on physician diagnosis and chart notes for diagnosis is likely to be more accurate than cross-sectional diagnostic interviews.

However, this study has several weaknesses. Generalizability may be limited, as patients in this study were drawn from highly specialized clinics. Also, many EM patients at the headache clinic are likely to have had CM in the past, which would reduce differences between groups. We did not differentiate between types of epilepsy or account for the presence of migraine aura. We lacked a validated “ability to work” scale that would apply to both migraine and epilepsy, and would account for people who are homemakers or have stopped trying to work. Our disability and impact scales overlap in what they measure, however the correlations between these scales were modest, indicating diversity in what they measured. Other interesting data, such as duration of illness, psychiatric co-morbidity, number of medications, and body mass index, were not collected.

Stigma has important public health implications that ought to be addressed [6]. As in epilepsy, we should undertake to reduce the stigma of migraine. These interventions may take place at multiple levels: by endeavoring to reduce stigma among the public through education, advocacy, and legal and policy interventions; at the organizational level, through training programs for clinicians; and at the intrapersonal level, through counseling, therapy, support, and empowerment programs [16].
